# Charting normative reference values and *Z*-scores for MRI-derived in vivo placental growth

**DOI:** 10.1007/s00247-025-06469-y

**Published:** 2025-11-15

**Authors:** Marin Jacobwitz, Julius Ngwa, Kushal Kapse, Catherine Limperopoulos, Nickie Andescavage

**Affiliations:** Children’s National Health System, 111 Michigan Ave NW, Washington, DC 20010 United States

**Keywords:** Placenta, Placental volume, *Z*-score, Magnetic resonance imaging (MRI)

## Abstract

**Background:**

In vivo placental volume derived from magnetic resonance imaging (MRI) is a novel imaging tool to evaluate the placenta during pregnancy, as the placenta is difficult to access throughout gestation. There is a paucity of established standardized normative raw values and *Z*-scores for in vivo placental volume based on MRI.

**Objective:**

To establish normative references for in vivo placental MRI-based volumes derived from a large cohort of healthy pregnant women carrying healthy fetuses throughout gestation.

**Materials and methods:**

Healthy pregnant women with healthy singleton pregnancies greater than 16 weeks gestation were enrolled in a longitudinal, prospective observational study. In total, 313 placental MRIs were analyzed from 209 pregnant women. In-vivo placentas were manually segmented to derive volumes and *Z*-scores. Means, standard deviations, and percentiles for normative reference raw values were calculated using weekly gestational age (GA) bins. Placental volume *Z*-scores were calculated based on 2-week GA bins using means and standard deviations.

**Results:**

Normative reference placental volumes from 209 subjects (313 scans) with median GA 31.43 [8.86] weeks are presented in weekly and bi-weekly GA bins. Using 2-week GA intervals, 95% of placental volume *Z*-scores were within ±2 standard deviations of the population mean.

**Conclusion:**

This data provides established normative in vivo raw and *Z*-score values derived from placental MRI. The value of accessing the placenta in vivo through MRI has become increasingly recognized, as the importance of the placenta in fetal and postnatal health is now more widely known. Establishing normative reference values for the in vivo placenta throughout gestation benefits both the clinical and scientific communities.

**Graphical abstract:**

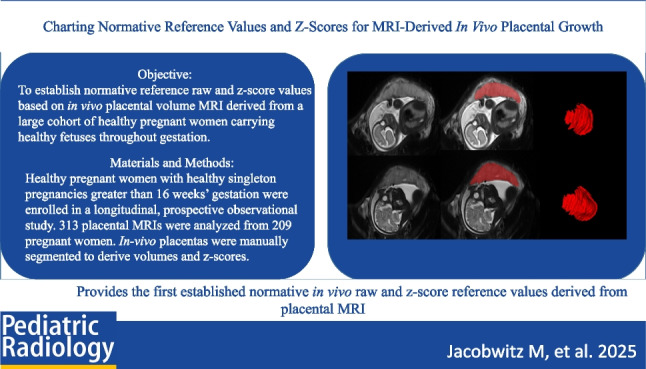

**Supplementary Information:**

The online version contains supplementary material available at 10.1007/s00247-025-06469-y.

## Introduction

The placenta is essential for normal fetal growth, acting as the interface between maternal and fetal circulations to ensure adequate nutrient and oxygen delivery to the fetus [1, 2]. It is well established that placental health is important for a successful pregnancy, as placental and umbilical cord abnormalities have been associated with adverse pregnancy and birth outcomes, including an increased risk for stillbirth [3–5]. However, more recently, the Developmental Origins of Health and Disease (DOHaD) theory has shown that the placenta is implicated in the longitudinal health of an individual throughout their lifespan through a process known as fetal programming [5–10]. The placenta is now recognized as a vital contributor to postnatal pediatric- and adult-onset chronic disease [4, 7–10]. Understanding placental health during gestation has renewed importance, given that the optimal health of this transient organ is essential for lifelong health [7].

Non-invasive imaging techniques to evaluate the placenta are important to monitor placental health throughout pregnancy. 2D ultrasonography remains the standard of care for pregnancy monitoring, as it is widely accessible and used predominantly for fetal evaluation and placental positioning [11, 12]. However, ultrasonography has technical limitations influenced by factors such as maternal body habitus, which can impact the depth of insonation [12]. Furthermore, abdominal wall adipose tissue can adversely influence the absorption of sonographic waves [12]. Magnetic resonance imaging (MRI) is a safe alternative to ultrasonography that offers unprecedented access to the in vivo placenta, providing the opportunity to identify placental abnormalities in both healthy and high-risk pregnancies throughout gestation [13–15]. Although placental MRI is gaining increased interest, there remains a paucity of normative reference values for in vivo placental volume throughout gestation based on MRI, which is important to provide control data for comparison to both healthy and high-risk populations, as well as to identify potential placental growth abnormalities.

The primary aim of this work was to establish a descriptive nomogram of placental volume based on in vivo placental volume MRI derived from a large cohort of healthy pregnant women carrying healthy fetuses throughout gestation that can serve as necessary reference values from which to detect early deviations from typical development. Secondary aims were to explore the effect of potential covariates on this normative dataset that have been shown to impact the maternal-fetal environment and placental development.

## Materials and methods

### Patient population

Subjects with singleton pregnancies greater than 16 weeks gestation were enrolled in a longitudinal, prospective observational study from March 2014 to November 2024. Participants were healthy volunteers with no significant pregnancy-related and/or chronic medical conditions, normal prenatal screening, anatomical imaging, and subsequent normal neurodevelopmental testing. Exclusion criteria were multiple gestation pregnancies, maternal contraindications to MRI, known/suspected congenital infections, and/or documented chromosomal abnormalities. Participants with structural abnormalities, fetal demise, or postnatal genetic diagnoses were excluded. The study was approved by the institutional review board. Written informed consent was obtained and HIPAA compliance was maintained for all participants.

### Study design

This was a retrospective analysis of prospectively collected data (described above). The research protocol included up to two serial fetal MRIs based on gestational age (GA) at the time of enrollment and participant availability.

### Fetal MRI

Studies were performed on a 1.5-T Discovery MR450 Scanner (83%, 261/313; GE Healthcare, Milwaukee, WI) using an eight-channel surface receive coil (USAI, Aurora, OH) or a 3.0-T Siemens MAGNETOM Vida Scanner (17%, 52/313; Siemens Medical Solutions, Malvern, PA) using up to a 48-channel surface receive coil system. For 1.5 T, single-shot fast spin echo (SSFSE) T2-weighted images were performed as follows: fat suppressed with TE 160 ms, TR 1100 ms, FOV 420×420 mm, 4-mm slice thickness, 0-mm slice gap, and 40 to 60 consecutive slices for full placental coverage in the axial plane. For 3.0 T, half-Fourier acquisition single-shot turbo spin-echo (HASTE) T2-weighted images were performed as follows: fat suppressed with TE 107 ms, TR 1500 ms, FOV 420×420 mm, 4-mm slice thickness, 0-mm slice gap, and 40 to 60 consecutive slices for full placental coverage in the axial plane. Interleaved acquisition, including odd and even slices, was performed to avoid slice cross-talk. Scanning protocols were harmonized between the 1.5-T and 3.0-T scanners to ensure consistency. No contrast or sedation was used.

### Post-processing: image segmentation and volume calculations

**Fig. 1:**
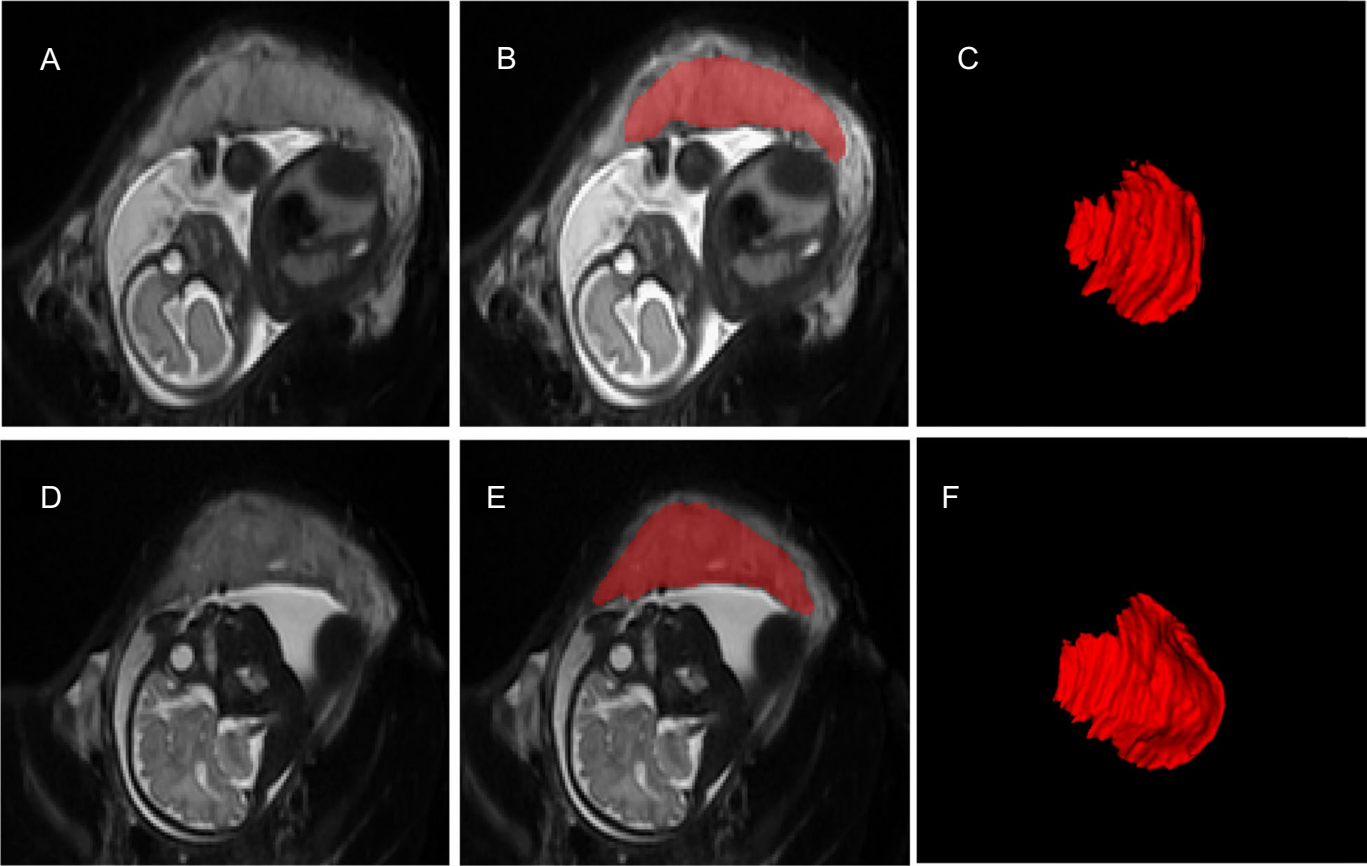
In vivo placental volume segmentation of a fetus at ~27 weeks gestation (**a**-**c**) and the same fetus at ~35 weeks gestation (**d**-**f**) depicting the placenta in the axial plane (**a**, **d**), in the axial plane with manual segmentation (**d**, **e**), and the 3-D reconstruction (**c**, **f**)

Placental segmentations were manually performed by one of three trained scientists. The placenta-uterine interface was identified via change in signal intensity using the adjacent uterine wall for guidance and manually outlined in the axial plane and verified in sagittal and coronal planes using ITK-SNAP software [16]. The volume was converted from cubic millimeters to cubic centimeters (Fig. 1). Intra-rater and inter-rater reliability were measured via intraclass correlation coefficient based on 75% of the cohort (235 scans).

Although there are multiple open-source automatic segmentation tools that have been used with varying success [17], there remain many challenges specific to placental imaging. Greater variability in the position and shape of the placenta compared to other organs and deformation that can occur with fetal movement, as well as implantation of the placenta into the myometrium that can obscure the uterine/placental border, are unique challenges to automated placental segmentation. Higher maternal body mass index and/or late gestational age scans contain higher imaging noise, further impacting the contrast between placenta/myometrium. Thus, manual segmentation minimizes errors in placental segmentation and remains the gold standard.

### Demographic and clinical data

Clinical and demographic data were collected for each subject. GA was calculated based on the last menstrual period or first-trimester ultrasound. Potential covariates of interest were explored relative to placental volume, including maternal age [18], parity [19], education, and fetal sex [19]. As maternal socio-economic status has been associated with abnormal placentation and adverse birth outcomes [20–22], maternal education was used as a proxy for socio-economic status in this work.

### Statistical analysis

Descriptive statistics for normally distributed continuous demographic and clinical measures, among all 209 subjects, were reported as mean (standard deviation), while non-normally distributed measures were reported as median (interquartile range). Categorical data were reported as count (percentages). Categorical variables (fetal sex, primiparous versus multiparous) were based on the total subject number (*N*=209), as these variables do not change between scans for the same subject. Continuous variables (placental volume, maternal age at MRI scan, fetal GA at MRI scan) were based on the total scan number (*N*=313). Given the significant influence of gestational age at MRI, our decision to use each placental MRI scan as an individual data point is based upon the observation that the subsequent value will be sufficiently different given the time interval between acquisition. As such, individual data observations (MRI scans) from distinct gestational ages within the same subject would provide sufficient variability in the data, as the placenta evolves throughout gestation. Of those with two scans, the interval range between acquisitions was 3-15 weeks. Normative values in the cohort of healthy women with healthy fetuses were reported by weekly GA bins. Weekly GA windows were created from 18 weeks to 40 weeks representing GA at the time of the MRI scan. For each weekly GA window, the counts, mean, standard deviation, and 10th, 25th, 50th, 75th, and 90th percentiles were reported as normative placental volume (cm^3^) reference values. Due to sparse data in weekly bins, bi-weekly GA bins were also created to compute *Z*-scores of placental volumes (cm^3^), with the initial bin comprising less than or equal to 20 weeks gestation due to scarce imaging in the early second trimester. Raw placental volumes demonstrate a fairly normal distribution utilizing histogram visual inspection. *Z*-score groupings were created ranging from below -1 to above 1, and based on each scan representing an individual data point (*N*=313; Online Resource 1). Descriptive statistics of placental volume relative to maternal age at scan, maternal parity, maternal education, and fetal sex were reported by *Z*-score groupings. Statistical analyses were conducted using the Rstudio 2024.12.1 build 563 software.

## Results

**Fig. 2:**
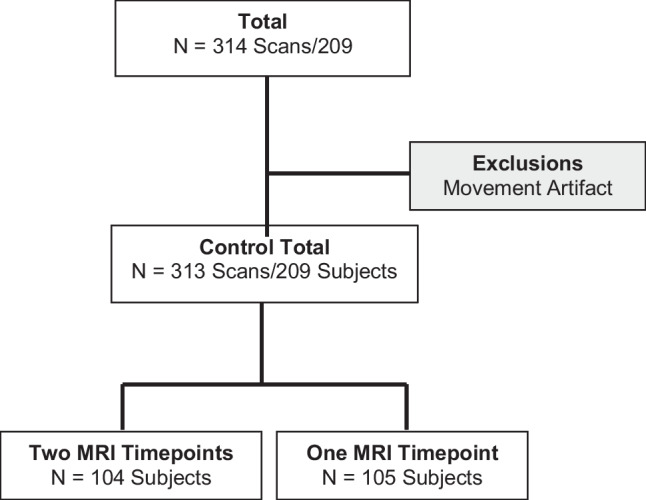
Flow-chart depicting the final cohort (subjects and scans), including exclusion with justification and the number of MRIs (one or two timepoints) per subject

A total of 210 pregnant women were enrolled between 16 weeks and 40 weeks gestation, of which 104 women had two fetal MRIs for a total of 314 scans. Of these, one subject was excluded due to excessive motion rendering segmentation impossible. The final cohort comprised 209 pregnant women (313 scans); 104 had two MRIs during their pregnancy (Fig. 2). The inter-rater reliability for placental segmentations was 0.97 (0.95-0.98) and intra-rater reliability was 0.92 (0.81-0.96).

The demographics of the cohort are in Table 1. In total, 49% (103/209) of the cohort were male fetuses. The majority of the cohort comprised non-Hispanic white women (41%, 85/209), followed by non-Hispanic black women (35%, 74/209) based on maternal self-reported race/ethnicity (Table 1). Using maternal education as a proxy for socio-economic status, most of the cohort had some secondary education (39%, 82/209) or graduate professional training (37%, 77/209; Table 1). This was the first pregnancy for 28% (57/209) of the enrolled pregnant women (Table 1).

**Table 1:** Demographics

Variable	*N*=209 subjects
Sex of fetus	
Male	103 (49%)
Female	106 (51%)
Maternal race^a^	
Asian/Pacific Islander	12 (6%)
Black, not of Hispanic origin	74 (35%)
Hispanic	20 (10%)
White, not of Hispanic origin	85 (41%)
Other	13 (6%)
Maternal education level^b^	
High school graduate or less	38 (18%)
Any secondary education	82 (39%)
Graduate professional training	77 (37%)
Maternal age at scan (years)^c^	31.69±6.79
Maternal parity^d^	
Primiparous	57 (28%)
Multiparous	146 (72%)
Gravida^e^	2.41±1.46
Fetal GA at MRI scan overall (weeks)	28.46±5.18
Fetal GA at MRI scan 1 (weeks)	28.10±5.08
Fetal GA at MRI scan 2 (weeks)	35.39±2.45
Placental volume (cm^3^) overall	617.80±313.84
Placental volume (cm^3^) at scan 1	585.52±262.51
Placental volume (cm^3^) at scan 2	910.27±333.91

Continuous measures=mean (SD); categorical measures=*N* (%)

^a^Data available for 97% (305/313) scans; 97% (204/209) subjects

^b^Data available for 95% (297/313) scans; 94% (197/209) subjects

^c^Data available for 98% (308/313) scans; 98% (204/209) subjects

^d^Data available for 97% (304/313) scans; 97% (203/209) subjects

^e^Data available for 96% (302/313) scans; 97% (203/209) subjects

**Fig. 3:**
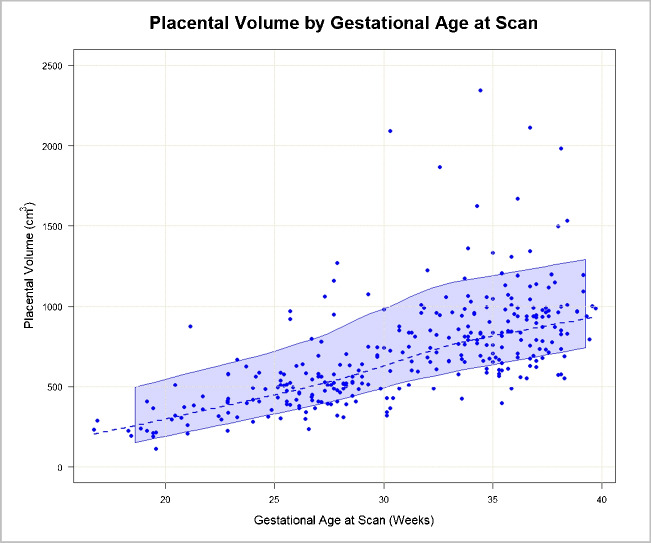
Scatterplot with confidence interval bands depicting raw placental volumes across gestational ages demonstrating how this normative dataset represents each trimester, with the majority of scans occurring within the second and third trimesters

Using 1-week GA intervals, placental volume means, standard deviations, and percentiles are presented in Table 2, representing normative values in a cohort of healthy women carrying healthy fetuses. Raw placental volumes based on 1-week GA intervals are depicted in Fig. 3.

**Table 2:** Normative placental volume reference values

Percentiles								
Gestational age (weeks)	*N*	Mean	SD	10	25	50	75	90
Below 18	2	261.06	41.00	237.87	246.57	261.06	275.56	284.25
18-19	4	223.60	22.18	201.76	216.59	231.25	238.27	239.31
19-20	7	246.98	102.64	159.63	201.44	216.34	294.93	381.63
20-21	5	360.36	87.73	299.99	306.88	319.17	372.11	453.79
21-22	6	420.72	238.38	243.23	283.89	370.60	424.46	657.33
22-23	9	396.80	120.94	281.66	316.94	402.55	426.79	577.13
23-24	4	500.46	174.51	335.34	376.01	513.15	637.60	655.43
24-25	10	426.25	103.72	311.11	355.03	412.86	491.80	566.14
25-26	17	531.85	172.24	389.65	434.08	506.53	538.24	721.11
26-27	19	451.40	138.11	298.16	367.21	436.70	505.08	631.96
27-28	24	601.70	257.64	398.19	408.41	516.69	609.32	1028.50
28-29	18	513.74	95.29	402.10	468.94	518.68	555.61	634.02
29-30	11	654.24	169.86	512.47	519.70	638.96	720.81	747.78
30-31	14	710.60	452.21	347.42	427.84	656.76	824.97	949.47
31-32	11	778.41	170.79	595.50	629.94	811.43	898.51	992.01
32-33	13	893.61	353.38	617.04	681.07	822.41	961.48	1190.20
33-34	21	824.80	210.54	653.31	670.22	803.71	943.33	1066.71
34-35	20	936.75	400.13	656.95	742.59	834.12	968.17	1114.78
35-36	29	825.78	243.32	577.25	645.95	817.05	1,008.20	1,148.57
36-37	23	958.22	362.31	638.68	699.68	938.19	1,017.75	1,312.27
37-38	24	891.56	161.59	688.21	767.57	913.71	974.34	1,132.54
38-39	16	971.71	391.28	574.28	723.37	907.33	1,000.19	1,513.84
39-40	6	1,002.30	136.47	867.65	951.71	993.75	1,071.21	1,145.50

Using 2-week GA intervals, 95% of placental volume *Z*-scores were within ±2 standard deviations of the population mean (Online Resource 2). Characteristics of placental volume *Z*-scores and covariates of interest are presented in Table 3.

**Table 3:** Characteristics of participants by placental volume *Z*-scores

Characteristics		Below –1 *n*=27 scans	–1 to –0.5 *n*=74 scans	–0.5 to 0 *n*=86 scans	0 to 0.5 *n*=61 scans	0.5 to 1 *n*=32 scans	Above 1 *n*=33 scans
Age at scan – weeks^+^		30.52 (5.66)	30.91 (5.40)	29.74 (5.88)	32.35 (4.94)	31.54 (5.42)	29.97 (5.78)
Maternal age at scan – years^+^		29.58 (6.25)	32.74 (6.18)	31.22 (6.10)	30.66 (7.68)	32.04 (7.13)	36.31 (5.58)
First pregnancy*	No	10/16 (62%)	33/48 (69%)	48/63 (76%)	24/31 (77%)	13/22 (59%)	18/23 (78%)
	Yes	6/16 (38%)	15/48 (31%)	15/63 (24%)	7/31 (23%)	9/22 (41%)	5/23 (22%)
Fetal sex*	Male	12/18 (67%)	28/49 (57%)	29/64 (45%)	16/33 (48%)	11/22 (50%)	7/23 (30%)
	Female	6/18 (33%)	21/49 (43%)	35/64 (55%)	17/33 (52%)	11/22 (50%)	16/23 (70%)

Continuous measures=mean (SD); categorical measures=*N* (%)

^+^*N*=313 scans

*N=209 subjects

## Discussion

In this study, we generated a large cohort of normative in vivo placental volumes based on MRI to provide normative reference values with percentiles and *Z*-scores. As the placenta is sensitive to changes based on maternal and fetal health, this normative cohort consists only of healthy pregnant women with no known pre-existing or pregnancy-associated comorbidities carrying a healthy fetus. Having access to normative values for in vivo placental measurements is valuable as the scientific community continues to explore the impact of the placenta on fetal and postnatal health in the previously inaccessible in vivo placenta.

Abnormalities in placental growth and function have potentially dire consequences for the fetus in utero [2, 7, 9, 10], with approximately 10-20% of in-utero fetal demise due to umbilical cord and placental complications [23]. In a study of placental pathology from healthy pregnancies that resulted in full-term healthy neonates, 78% of placentas had acute or chronic inflammatory lesions or evidence of malperfusion, emphasizing that subtle alterations of disturbed placental growth may go unnoticed [24]. However, the placenta is not a static organ, adapting to the in-utero environment based on the availability of maternal nutrients and oxygen to promote fetal development despite hostile conditions [2]. Despite increased interest in the placenta, it remains the most poorly understood organ given the accessibility limitations during pregnancy [25]. For this reason, a majority of studies on the placenta, including standard measures of placental weight and placental pathology, are at the time of birth when the placenta is expelled and the neonate is born [25–31]. The difficulty with this approach is that adverse pregnancy complications that may impact fetal health and development are only recognized postnatally, limiting the opportunity for perinatal, maternal, or fetal intervention to potentially heed off lifelong consequences for the child.

Understanding placental volumetry in low-risk pregnant women with healthy fetuses provides important reference values for high-risk pregnancies impacted by maternal and/or fetal comorbidities. Disrupted placental growth has long been associated with pregnancy complications, such as intrauterine growth retardation (IUGR), preeclampsia, and gestational diabetes [32–35], though these studies have almost exclusively relied on pathologic assessments of placental weight *after* delivery. Similarly, chronic placental insufficiency is only suspected *after* the emergence of fetal growth restriction [36], at a point where placental failure has already negatively impacted fetal growth and development. Often, surrogate measures of placental health such as Doppler evaluation of the umbilical vessels are utilized with varying sensitivity and specificity [37, 38]. To better prevent complications of disrupted placental growth, real-time, reliable, and in vivo measures of placental development are needed. Although some studies have successfully utilized three-dimensional (3-D) ultrasonography to evaluate placental volumetry as a predictor for small-for-gestational-age infants and adverse perinatal outcomes [32–34], routine prenatal ultrasound evaluation of the placenta focuses predominantly on its location relative to the cervical os to evaluate for implantation disorders (i.e., accreta or previa) [33]. Maternal body habitus, fetal and placental position, and other clinical characteristics provide additional challenges for placental evaluation via ultrasound [12, 39, 40]. Additionally, placental volume measured via two-dimensional sonography has shown poor correlation with MRI [41] and, although there are emerging automatic segmentation pipelines being explored [39, 40], ultrasonography remains largely operator dependent [37].

Beyond assessments of implantation disorders, in vivo placental MRI provides an opportunity to assess the placenta throughout gestation, with the future goal of identifying placental dysfunction early enough that there is an opportunity to intervene. Abnormal placental volumetry has been detected via MRI and associated with congenital heart disease [42], fetal growth restriction [43, 44], and adverse pregnancy outcomes (severe preeclampsia, abnormal antenatal surveillance, perinatal mortality) [45]. The availability of normative placental reference values opens the opportunity for early detection of impaired placental development. Ideally, detection of altered placental development prior to maternal-fetal consequences will allow for novel interventions to augment placental function and carefully manage delivery conditions if and when appropriate, with the goal of providing optimal birth outcomes. This study adds to the existing literature [14, 46, 47] on normative placental MRI volumetry with a large cohort prospectively recruited to ensure healthy participants, thereby establishing a large dataset of normative in vivo placental volume reference values to further the field of in vivo placental MRI.

### Limitations

Several limitations need to be acknowledged. First, given the protocol design with recruitment of pregnant women at or greater than 16 weeks gestation, data available for the earlier weekly GA intervals were limited. Second, given the small sample of first and second trimester scans, we did not have sufficient scan numbers to generate weekly GA *Z*-scores. Therefore, our *Z*-scores are based on 2-week GA bins and are overall a representation of the latter half of gestation. Third, our cohort was overall well educated and predominantly comprised non-Hispanic white or non-Hispanic black women. Future studies should include cohorts with more racial and ethnic diversity and individuals of lower socio-economic status to increase generalizability. Fourth, although we were able to collect demographic data on >90% of participants, there were instances of missing data, which are clearly noted in the table legends. Lastly, it should be emphasized that the intention of this paper was to solely perform a descriptive analysis of our normative dataset. Therefore, no statistical inference on associations should be drawn from our analyses. Future studies should aim to acquire a larger sample of placental volume data in healthy women pregnant with healthy fetuses to continue to establish true normative data, particularly within the first and second trimesters; however, it should be acknowledged that this is challenging, as most pregnant women do not receive their first prenatal ultrasound until approximately 11 weeks gestation. In addition, future studies should continue to explore and refine placental automatic segmentation pipelines to increase uniformity and speed of data processing and analyses across sites.

## Conclusion

In this study, we generated normative placental volumes and placental volume *Z*-scores to be used as normative reference values. Clinicians and scientists alike can utilize this data for comparison with both their own normative data and as control data for high-risk populations as investigations continue into the in vivo placenta and the utility of placental MRI becomes more widely accepted.

## Supplementary Information

Below is the link to the electronic supplementary material.

Supplementary file1 (DOCX 21.9 KB)

## Data Availability

Data is available upon request from the corresponding author.
