# Supplementary material for: Charting normative reference values and Z-scores for MRI-derived in vivo placental growth
Source: Pediatr Radiol. 2025 Nov 15;56(2):384–92. doi: 10.1007/s00247-025-06469-y (PMC12881100; doi:10.1007/s00247-025-06469-y)
Supplement: Supplementary file 1 — Supplementary file1 (DOCX 21.9 KB) [file 247_2025_6469_MOESM1_ESM.docx]

**Online Resource 1. Number of MRI Scans Comprising 2-week GA Intervals for Z-Score Derivation**

| **Bins** | **Number of Scans** |
| --- | --- |
| </= 20 weeks | 13 |
| 20-22 weeks | 11 |
| 22-24 weeks | 16 |
| 24-26 weeks | 26 |
| 26-28 weeks | 43 |
| 28-30 weeks | 29 |
| 30-32 weeks | 25 |
| 32-34 weeks | 34 |
| 34-36 weeks | 48 |
| 36-38 weeks | 49 |
| 38-40 weeks | 19 |

**Online Resource 2. Normative Analysis of Placental Volumes among Healthy Pregnant Women**

**
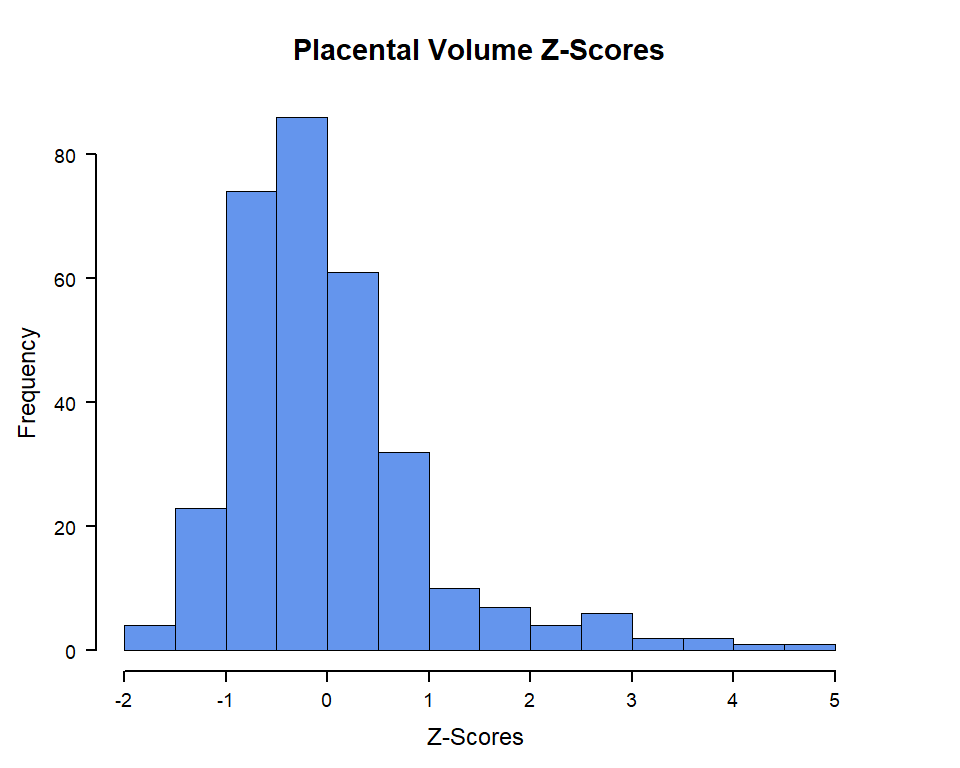
**
